# Trends, Hotspots, and Future Directions of Research on Pain in Dystonia: A Bibliometric Analysis

**DOI:** 10.1002/brb3.70959

**Published:** 2025-10-15

**Authors:** Xiaonan Liu, Bin Wu

**Affiliations:** ^1^ Department of Anesthesiology The First Affiliated Hospital of Chongqing Medical University Yuzhong District China

**Keywords:** bibliometrics, botulinum toxin, CiteSpace, dystonia, pain

## Abstract

**Introduction:**

Pain in dystonia is closely related neurological disorders, with significant impact on patients' quality of life. This study aims to conduct a bibliometric analysis to examine the development trends, research hotspots, and future directions in the field of pain in dystonia.

**Methods:**

Publications from 1981 to 2025 were retrieved from the Web of Science Core Collection database. Bibliometric data were analyzed using the R package “Bibliometrix,” VOSviewer, and CiteSpace.

**Results:**

A total of 1225 articles were included in this study. The USA had the highest publication volume (329), followed by Germany (103) and Italy (87). The most productive institution was the University of London with 123 publications. *Movement Disorders* was one of the most influential journals in its field. Key authors in the field included Jankovic Joseph, Marinus Johan, and Van Hilten Jacobus J. The high‐frequency keywords were “double‐blind,” “efficacy,” and “spasmodic torticollis.” Keywords burst analysis showed emerging interests in “blepharospasm,” “botulinum neurotoxin,” “quality of life,” “diagnosis,” “management,” and “neurotoxin.”

**Conclusion:**

This bibliometric study quantitatively analyzed research trends in pain in dystonia, identifying key contributors, hotspots, and emerging trends. Keywords result reflected the growing interest in improving patient outcomes through better diagnostic techniques and therapeutic interventions aimed at alleviating symptoms and enhancing the quality of life for individuals affected by this condition.

## Graphical abstract

This bibliometric study, based on 1225 articles on pain in dystonia published between 1981 and 2025 within the Web of Science Core Collection, reveals key trends in the field. The research employed multiple bibliometric analysis tools, including the R package “Bibliometrix,” VOSviewer, and CiteSpace. The findings reveal a shift in research focus from foundational concepts and therapeutic efficacy toward emerging topics, including diagnosis, management, quality of life, and the use of botulinum neurotoxin for treating dystonia‐related pain.

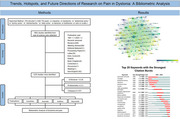

## 1 Introduction

Dystonia is a movement disorder characterized by sustained or intermittent muscle contractions, causing repetitive movements and abnormal postures (Thomsen et al. 2024). Dystonia can occur independently or alongside other movement disorders and neurological diseases, making it the third most common movement disorder (Grütz and Klein 2021). Cervical dystonia (CD) is the most frequently affected area, with a prevalence of 16.4 per 100,000 individuals (Stephen et al. 2023). Dystonia not only causes motor impairments, limb deformities, and reduced ability to perform daily activities, but also often leads to social isolation and emotional disturbances, such as depression, anxiety, and low self‐esteem (Frucht et al. 2020). Moreover, the motor disorder is accompanied by pain in most patients (Albanese et al. 2011), affecting between 55% and 90% of treated CD patients (Rosales et al. 2021), which is a source of considerable disability for patients (Rosales et al. 2021). Thus, management of pain in dystonia is an urgent issue around the world.

Pain is a common and multifaceted symptom experienced by individuals with dystonia, deeply affecting their quality of life (Listik et al. 2023). It often arises from excessive muscle contraction, spasms, and abnormal postures caused by dystonia, potentially involving areas such as the neck, back, and limbs (Rosales et al. 2021). The presence of pain not only exacerbates muscle tension and stiffness but also further limits motor function (Sterling et al. 2001). In addition, pain can lead to sleep disturbances, mood fluctuations, and social difficulties, significantly diminishing overall life quality (Alshehri et al. 2025). Furthermore, pain may reduce patient adherence to treatment plans, impacting treatment outcomes and creating a vicious cycle (Demartini et al. 2020). Given the ubiquity and severity of pain in individuals with dystonia, understanding the complex relationship between the two is crucial. Despite recognition of the pathophysiological mechanisms of dystonia and clinical advances, research on pain in dystonia remains limited, often focusing on specific aspects or patient groups. An integrated framework is needed to synthesize existing research and uncover current hotspots and potential frontiers.

Bibliometric analysis, a quantitative method for exploring research trends and knowledge domains, provides valuable insights into the global research landscape (Zhou et al. 2022a, Zhou et al. 2022b). Through visualization and statistical tools, bibliometric studies can identify influential contributors, collaboration networks, and emerging research hotspots. While such analyses have been conducted for other aspects of dystonia, including deep brain stimulation treatment for dystonia (Listik et al. 2020) and essential tremor (ET) in dystonia (King et al. 2016), there is a lack of comprehensive bibliometric evaluation specifically focused on pain in dystonia. To address this gap, the present study applies bibliometric methods to analyze global research trends on this field. This study aims to use bibliometric analysis to identify key contributors, research hotspots, and emerging themes in the field of pain in dystonia, providing guidance for future research directions.

## 2 Materials and Methods

### 2.1 Search Strategies and Data Collection

A comprehensive literature search was conducted using the Web of Science Core Collection (WoSCC) to investigate the pain in dystonia from January 1, 1981 (the earliest publication year in this field) to January 3, 2025. The WoSCC, a widely recognized and authoritative database, offers access to a wide range of high‐quality academic publications across various scientific fields. The search formula used was: (TS = (Dyston*)) AND TS = (pain∗ or migraine∗ or headache∗ or “abdominal ache∗” or “tummy ache∗” or “stomachache∗” or “belly ache∗” or earache∗ or toothache∗ or colic or sciatic∗) (Listik et al. 2020; Xiong et al. 2021). We adopted the broad search using above terms to ensure comprehensive coverage of relevant literature on various types of dystonia. Only publications in English were included, focusing solely on articles among various document types. To minimize discrepancies from database updates, the literature was retrieved on January 3, 2025. During the screening process, irrelevant document types such as reviews, meeting abstracts, and non‐English publications were excluded. The bibliographic information was exported using the “Full record and cited references” and “plain text” formats (Appendix). This information included publication and citation counts, titles, author details, institutions, countries/regions, keywords, and journals.

### 2.2 Statistical Analysis

Three bibliometric tools were used for visualization and in‐depth analysis: VOSviewer (version 1.6.20), CiteSpace (version 6.3.R1), and the R package “bibliometrix” (version 4.3.3).

VOSviewer was used for the visualization of collaborative networks, such as co‐occurrence of authors, countries, journals, keywords, and institutions, journal coupling network was also included, offering insights into the overall academic landscape of pain in dystonia research.

CiteSpace was employed to identify keyword bursts and emerging research trends in the field (Huang et al. 2021). The analysis was conducted using a time‐slicing parameter ranging from 1995 to 2024. Nodes were defined as keywords, with a threshold set for the top 5 keywords in each time slice. To ensure clarity in visualizing evolving trends, pruning was performed using the Pathfinder algorithm, and the networks were subsequently merged.

R package “Bibliometrix” was utilized for trend mapping and ranking analyses, enabling the tracking of publication and citation patterns among authors, institutions, and countries. This tool further facilitated the generation of trend charts and longitudinal analyses concerning the evolution of the research field (Zhao and Li 2023).

Various bibliometric indices, including the *h*‐index, *g*‐index, and *m*‐index, were employed to assess the academic impact of authors and journals. The *h*‐index measures both productivity and citation influence, reflecting the number of publications with at least h citations (Bertoli‐Barsotti and Lando 2017; Hirsch 2005; Ruscio 2016). The *g*‐index assigns more weight to highly cited works, while the *m*‐index normalizes the *h*‐index by an author's career length, offering a temporal perspective (Schreiber 2009). These metrics were derived from data exported from the WoSCC database. Journal quality and impact were assessed using Journal Citation Reports (JCR) quartile rankings and Impact Factor (IF). The JCR quartiles (Q1–Q4) categorize journals by their relative IF within specific disciplines, serving as benchmarks for academic influence (Dorta‐González and Dorta‐González 2013).

## 3 Results

### 3.1 Publication Trends

**FIGURE 1:**
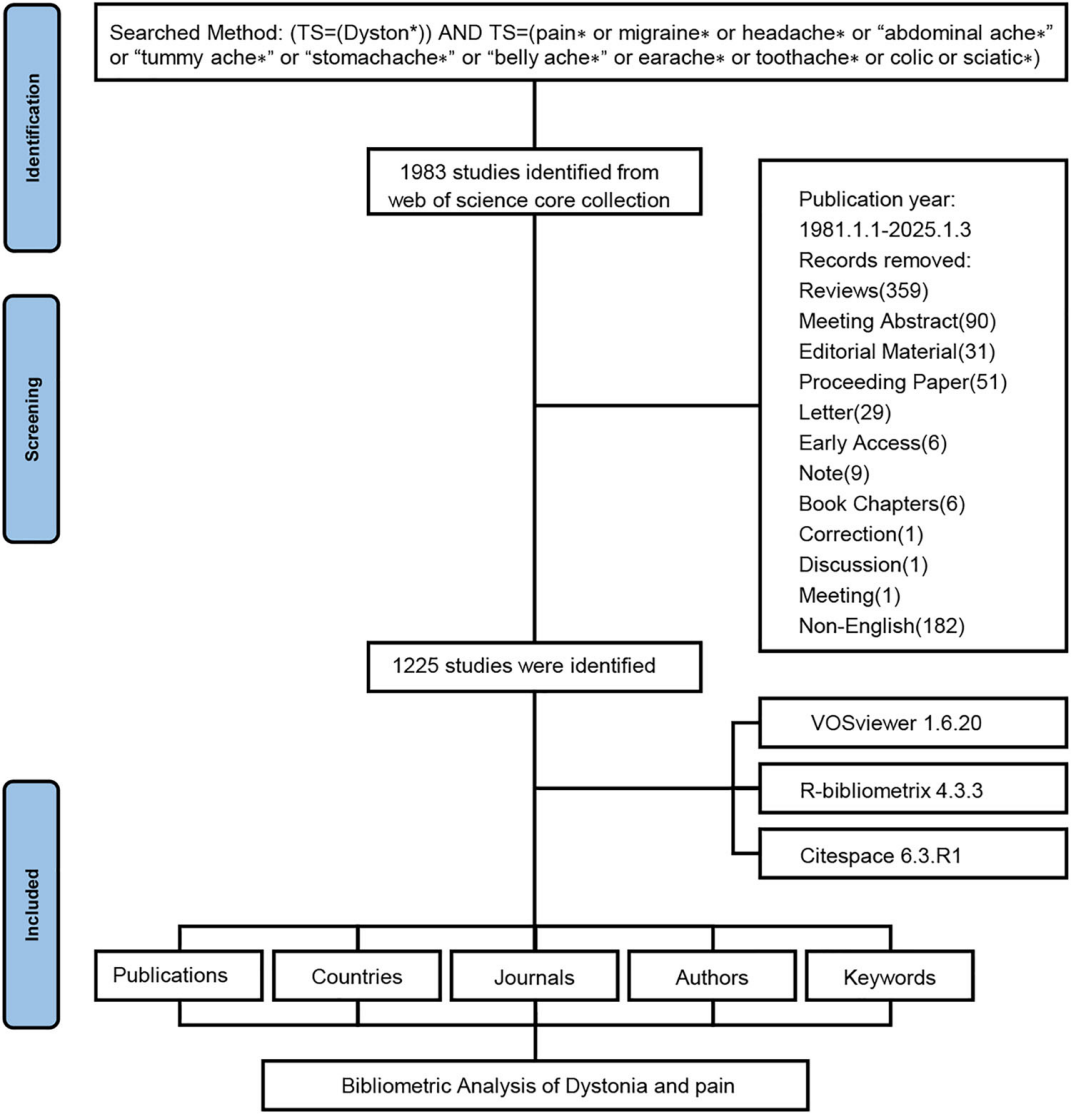
Flowchart of data screening process.

The data retrieval and collection protocol were shown in Figure 1. Our analysis encompassed 1225 peer‐reviewed articles published between 1981 and 2025, demonstrating an 8.01% annual growth rate that reflected the field's accelerating development. This multinational collaboration involved 5925 researchers from 4227 institutions across 63 countries/regions, with intellectual outputs disseminated through 415 academic journals and supported by 27,603 cited references.

**FIGURE 2:**
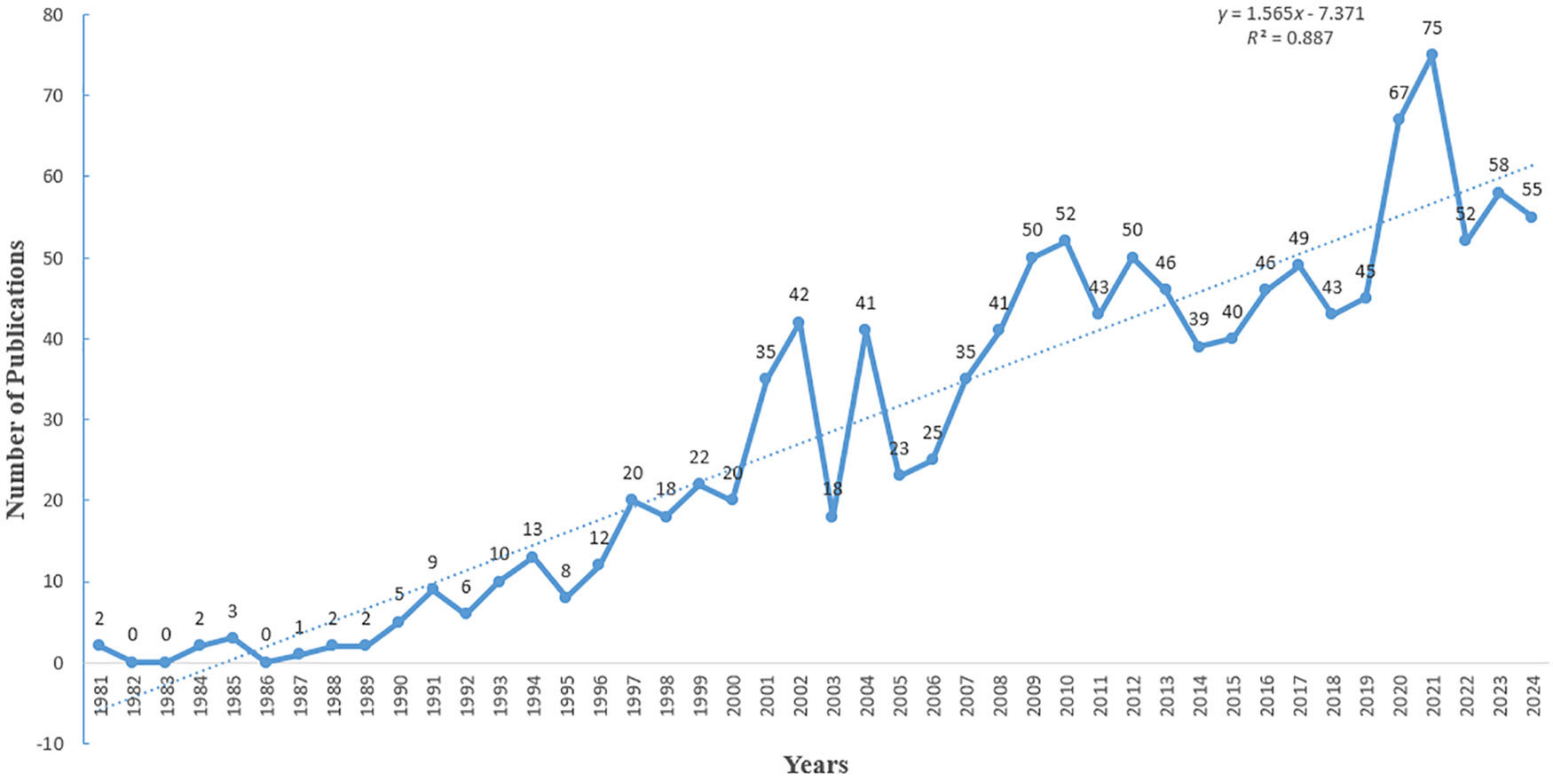
Annual number of publications from 1981 to 2024.

As shown in Figure 2, the publication trend revealed gradual growth over time, revealing three distinct phases: a nascent period (1981–1990) with annual outputs consistently below five publications; a transitional phase (1991–2000) marking gradual disciplinary maturation; followed by exponential growth post‐2000, culminating in peak productivity (*n* = 75) in 2021.

### 3.2 Analysis of Leading Countries

**FIGURE 3:**
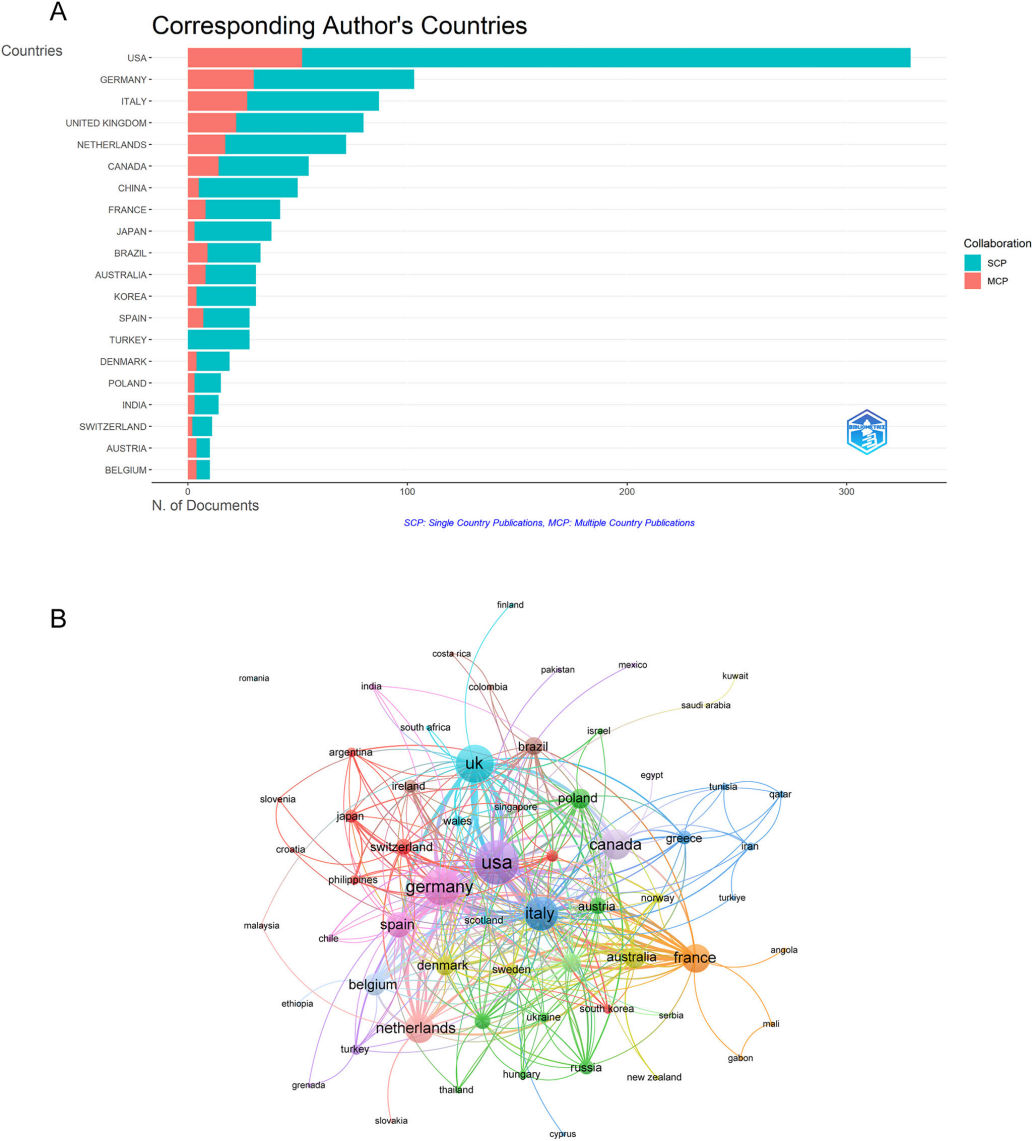
Global distribution and collaboration. (A) Distribution of corresponding authors' publications by country. The number of publications attributed to corresponding authors from different countries, distinguishing between single country publications (SCP) and multiple country publications (MCP). (B) Visualization map depicting the collaboration among different countries. The collaborative relationships between countries, with nodes representing countries, the size of nodes indicating publication count, and the thickness of links showing the strength of co‐authorship collaborations. Colors indicate different research clusters.

The global knowledge production landscape in this field exhibits concentrated geographical distribution with emerging multilateral cooperation patterns. As shown in Table 1, the USA led with 329 articles (26.86%), followed by Germany with 103 articles (8.41%) and Italy with 87 articles (7.10%). In terms of total citations (TC), the USA again took the lead with 14,220 citations, followed by Germany with 4236 citations and the United Kingdom with 2942 citations. Regarding total publications (TP), the USA maintained its leading position with 1131 publications, followed by Italy with 677 and Germany with 396. Furthermore, the USA contributed the most multiple country publications (MCP) with 52, followed by Germany with 30 and Italy with 27 (Figure 3). Among the 63 countries involved in international collaborations with at least one article, the USA (link strength = 2479) had the highest number of collaborations with other countries, followed by the United Kingdom (link strength = 182) and Germany (link strength = 174) (Figure 3B).

**TABLE 1:** Publication and citation profiles of leading countries.

Country	Articles	Freq	SCP	MCP	MCP‐ratio	TP	TP‐rank	TC	TC‐rank	Average citations
USA	329	26.857	277	52	15.81	1131	1	14,220	1	43.2
Germany	103	8.408	73	30	29.13	396	3	4236	2	41.1
Italy	87	7.102	60	27	31.03	677	2	2252	6	25.9
United Kingdom	80	6.531	58	22	27.50	319	4	2942	3	36.8
Netherlands	72	5.878	55	17	23.61	270	5	2278	5	31.6
Canada	55	4.490	41	14	25.45	219	7	1877	7	34.1
China	50	4.082	45	5	10.00	160	8	400	17	8
France	42	3.429	34	8	19.05	254	6	2421	4	57.6
Japan	38	3.102	35	3	7.89	99	11	741	9	19.5
Brazil	33	2.694	24	9	27.27	93	12	486	15	14.7
Australia	31	2.531	23	8	25.81	155	9	631	11	20.4
Korea	31	2.531	27	4	12.90	78	13	786	8	25.4
Spain	28	2.286	21	7	25.00	155	10	560	12	20
Turkey	28	2.286	28	0	0.00	60	15	243	21	8.7
Denmark	19	1.551	15	4	21.05	54	17	688	10	36.2
Poland	15	1.224	12	3	20.00	52	18	255	20	17
India	14	1.143	11	3	21.43	31	23	221	22	15.8
Switzerland	11	0.898	9	2	18.18	59	16	515	14	46.8
Austria	10	0.816	6	4	40	34	19	477	16	47.7
Belgium	10	0.816	6	4	40	60	14	174	25	17.4

*Note*: Articles: Publications of corresponding authors only.

Abbreviations: Average citations, the average number of citations per publication; Freq, frequency of total publications; MCP, multiple country publications; MCP‐ratio, proportion of multiple country publications; SCP, single country publications; TC, total citations of all authors; TC‐rank, rank of total citations; TP, total publications of all authors; TP‐rank, rank of total publications.

### 3.3 Analysis of Institutions

**FIGURE 4:**
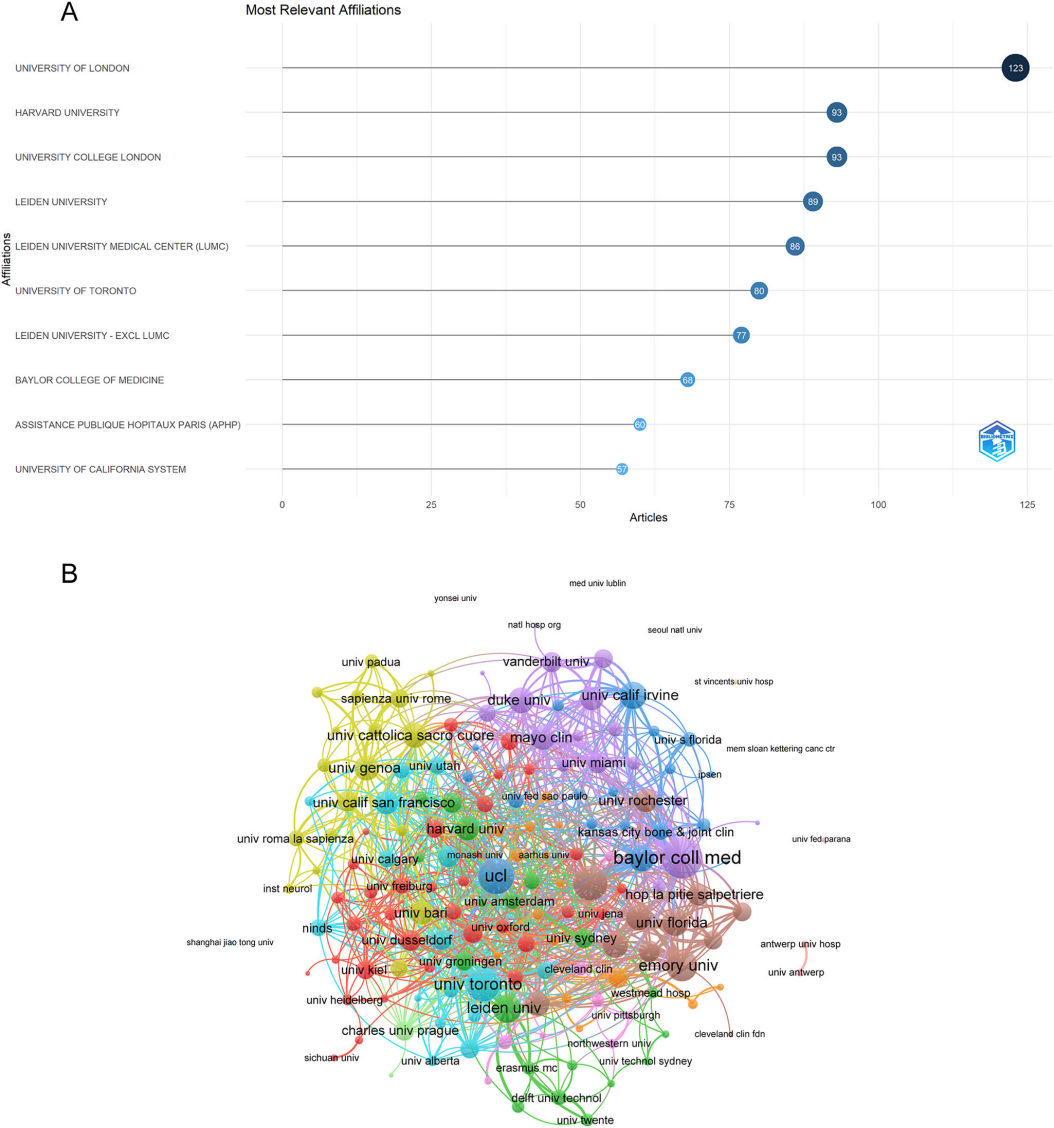
Institutional contributions and collaborations. (A) Top 10 institutions by article count and rank. The circle size shows the article count, with darker shades indicating higher ranks. (B) Visualization map depicting the collaboration among different institutions. Nodes represent institutions, with size indicating publication count. Links represent co‐authorships, with thickness showing collaboration strength of co‐authorship collaborations. Colors indicate different research clusters.

A total of 4227 institutions has contributed to the field of pain in dystonia research. The top 10 most productive institutions are displayed in Figure 4A. The University of London was the leading contributor, with 123 TP, followed by Harvard University and University College London, both with 93 TP. VOSviewer was used to visualize collaboration between institutions, as shown in Figure 4B. Among the 169 institutions involved in international collaborations with a minimum of four articles, Baylor College of Medicine had the highest number of collaborations (link strength = 123), followed by University College London (link strength = 93) and Rush University (link strength = 86).

### 3.4 Analysis of Authors

**FIGURE 5:**
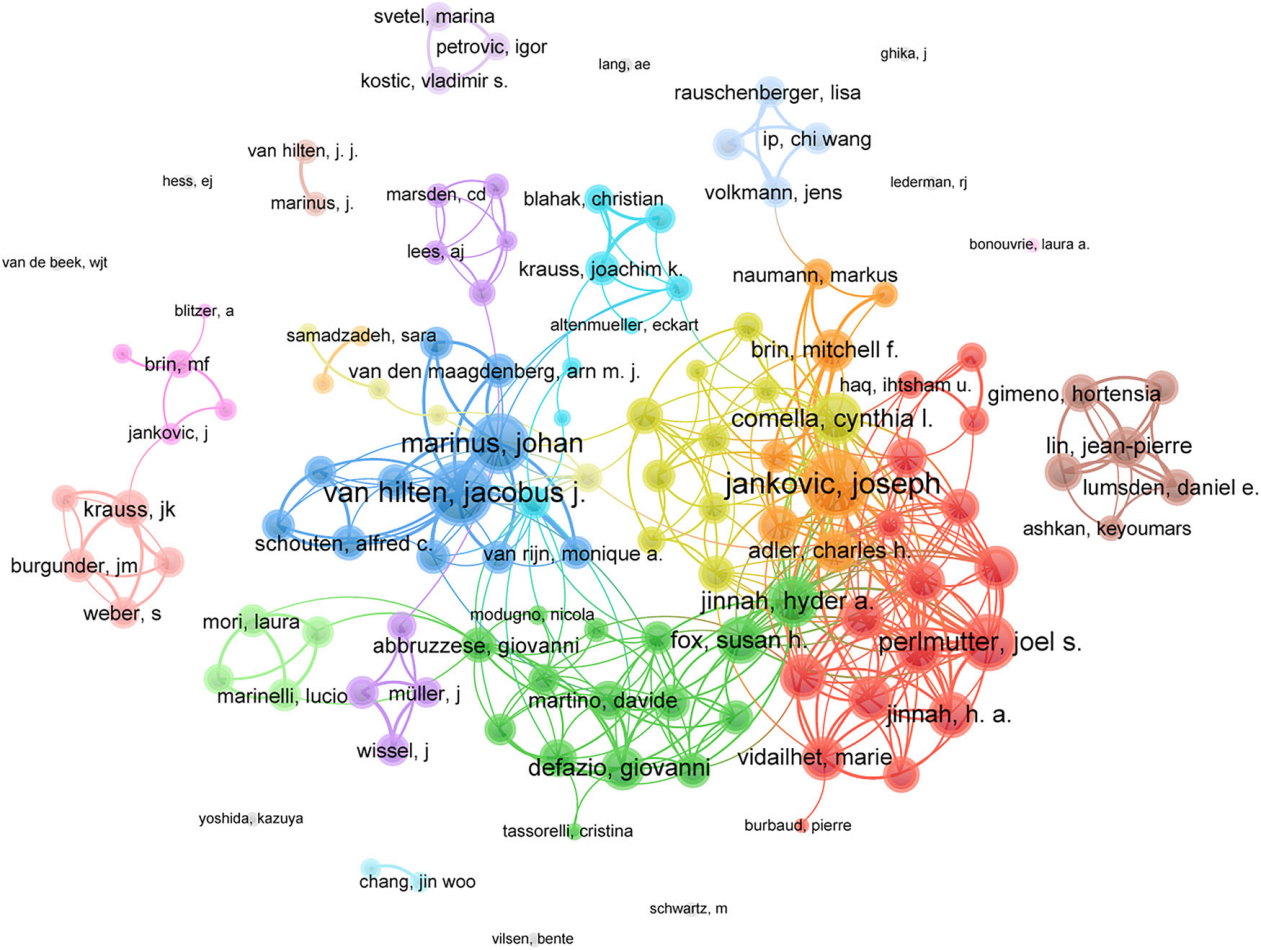
Author collaboration network. Visualization map depicting the collaboration among different authors. Nodes represent authors, with size indicating publication count. Links represent co‐authorships, with thickness showing collaboration strength. Colors indicate different research clusters. Link strength in collaboration networks measures the frequency of co‐authorship between authors, indicating the level of collaborative research.

A total of 5925 authors contributed to research in this field, demonstrating significant variations in productivity and impact. The information of top 20 authors who ranked by their *h*‐index was presented in Table 2. Jankovic Joseph emerged as the most influential researcher (*h*‐index = 26, followed by Marinus Johan and Van Hilten Jacobus J. (*h*‐indexes = 17 each). Jankovic Joseph ranked as the most prolific author with 38 TP, followed by Van Hilten Jacobus J. (31) and Marinus Johan (24). In term of TC, Jankovic Joseph also led with 3094 citations, surpassing Brin M. F. (1641) and Comella Cynthia (1083). The collaboration network, visualized in Figure 5, comprises 123 authors who have published at least four articles each. Jankovic Joseph demonstrated the strongest collaborative connections (link strength = 78), followed by Van Hilten Jacobus J. (link strength = 71) and Marinus Johan (link strength = 58).

**TABLE 2:** Publication and citation profiles of high‐impact authors.

Authors	*h*‐index	*g*‐index	*m*‐index	PY‐start	TP	TP‐Frac	TP‐rank	TC	TC‐rank
Jankovic Joseph	26	38	0.722	1990	38	10.78	1	3094	1
Marinus Johan	17	24	0.895	2007	24	4.09	3	708	8
Van Hilten Jacobus J.	17	29	0.895	2007	31	6.22	2	866	6
Brin M. F.	14	20	0.4	1991	20	2.7	5	1641	2
Comella Cynthia	13	19	0.722	2008	19	2.64	6	1083	3
Krauss J. K.	11	21	0.379	1997	21	3.95	4	909	4
Tinazzi Michele	11	14	0.579	2007	14	1.03	8	590	11
Albanese Alberto	10	15	0.556	2008	15	1.42	7	517	13
Bhatia Kailash P.	10	11	0.526	2007	11	1.49	10	753	7
Defazio Giovanni	9	14	0.5	2008	14	0.88	8	464	14
Modugno Nicola	8	9	0.4	2006	9	0.56	11	239	18
Poewe W.	8	8	0.235	1992	8	1.02	14	881	5
Burgunder J. M.	7	8	0.259	1999	8	1.99	14	650	9
Dressler Dirk	7	8	0.35	2006	8	2.05	14	165	20
Marsden C. D.	7	7	0.2	1991	7	1.73	18	543	12
Morgante Francesca	7	9	0.5	2012	9	0.58	11	259	17
Munts Alexander G.	7	7	0.389	2008	7	1.43	18	168	19
Tijssen Marina A. J.	7	9	0.368	2007	9	1.13	11	376	15
Van Den Maagdenberg Arn M. J. M.	7	8	0.412	2009	8	0.81	14	647	10
Van Rijn Monique A.	7	7	0.368	2007	7	1.43	18	332	16

*Note: h*‐index: The *h*‐index of the author, which measures both the productivity and citation impact of the publications. *g*‐Index: The *g*‐index of the author, which gives more weight to highly‐cited articles. *m*‐Index: The *m*‐index of the author, which is the *h*‐index divided by the number of years since the first published paper.

Abbreviations: PY‐start, publication year start, indicating the year the journal started publication; TC, total citations; TC‐rank, rank of total citations; TP, total publications; TP‐Frac, fraction of total publications; TP‐rank, rank of total publications.

### 3.5 Analysis of Journals

Research in this field spanned 415 academic journals, with the top 20 journals ranked by *h*‐index detailed in Table 3. *Movement Disorders* (Q1, IF = 7.4) emerged as the most influential journal, achieving the highest *h*‐index of 35. This was followed by the *Journal of Neurology, Neurosurgery and Psychiatry* (Q1, IF = 8.7, *h*‐index = 26), and *Neurology* (Q1, IF = 8.7, *h*‐index = 24). *Movement Disorders* also dominated in TP with 68, surpassing the *Journal of Neurology* (39) and *Frontiers in Neurology* (31). In term of TC, *Movement Disorders* maintained its leading position with 3062 citations, followed by *Neurology* (2629) and the *Journal of Neurology, Neurosurgery and Psychiatry* (1425).

**TABLE 3:** Bibliometric indicators of high‐impact journals.

Journal	*h*‐index	*g*‐index	*m*‐index	IF	JCR	PY‐start	TP	TP‐rank	TC	TC‐rank
*Movement Disorders*	35	57	1	7.4	Q1	1991	68	1	3062	1
*Journal of Neurology Neurosurgery and Psychiatry*	26	30	0.70	8.7	Q1	1989	30	4	1425	3
*Neurology*	24	30	0.67	7.7	Q1	1990	30	5	2629	2
*Journal of Neurology*	21	38	0.62	4.8	Q1	1992	39	2	707	6
*Parkinsonism & Related Disorders*	17	27	0.89	3.1	Q2	2007	28	6	573	8
*Pain*	16	20	0.50	5.9	Q1	1994	20	10	1027	5
*Brain*	15	15	0.45	10.6	Q1	1993	15	13	1056	4
*Headache*	14	19	0.33	5.4	Q1	1984	19	11	304	18
*European Journal of Paediatric Neurology*	13	22	0.76	2.3	Q2	2009	23	7	158	40
*Journal of Neural Transmission*	13	20	0.52	3.2	Q2	2001	20	9	230	24
*Journal of the Neurological Sciences*	12	19	0.36	3.6	Q2	1993	19	12	299	19
*Canadian Journal of Neurological Sciences*	11	14	0.27	2.9	Q2	1985	14	14	160	38
*Journal of Neurosurgery*	10	12	0.30	3.5	Q1	1993	12	21	531	9
*Developmental Medicine and Child Neurology*	9	11	0.38	3.8	Q1	2002	11	24	470	11
*European Journal of Neurology*	9	12	0.33	4.5	Q1	1999	12	19	411	13
*Frontiers in Neurology*	9	13	0.82	2.7	Q3	2015	31	3	157	41
*Neuromodulation*	9	11	0.41	3.2	Q2	2004	11	25	75	87
*Acta Neurologica Scandinavica*	8	8	0.20	2.9	Q2	1985	8	29	145	47
*Archives Of Physical Medicine and Rehabilitation*	8	11	0.27	3.6	Q1	1996	11	23	249	23
*Stereotactic and Functional Neurosurgery*	8	13	0.23	1.9	Q2	1991	13	17	207	27

*Note: h*‐index: The *h*‐index of the journal, which measures both the productivity and citation impact of the publications. *g*‐index: The *g*‐index of the journal, which gives more weight to highly‐cited articles. *m*‐index: The *m*‐index of the journal, which is the *h*‐index divided by the number of years since the first published paper. IF‐2023: Impact Factor in 2023, indicating the average number of citations to recent articles published in the journal. JCR‐2023: The quartile ranking of the journal in the Journal Citation Reports in 2023, indicating the journal's ranking relative to others in the same field (Q1: top 25%, Q2: 25%–50%, Q3: 50%–75%, Q4: bottom 25%).

Abbreviations: PY‐start, publication year start, indicating the year the journal started publication; TC, total citations; TC‐rank, rank of total citations; TP, total publications; TP‐rank, rank of total publications.

**FIGURE 6:**
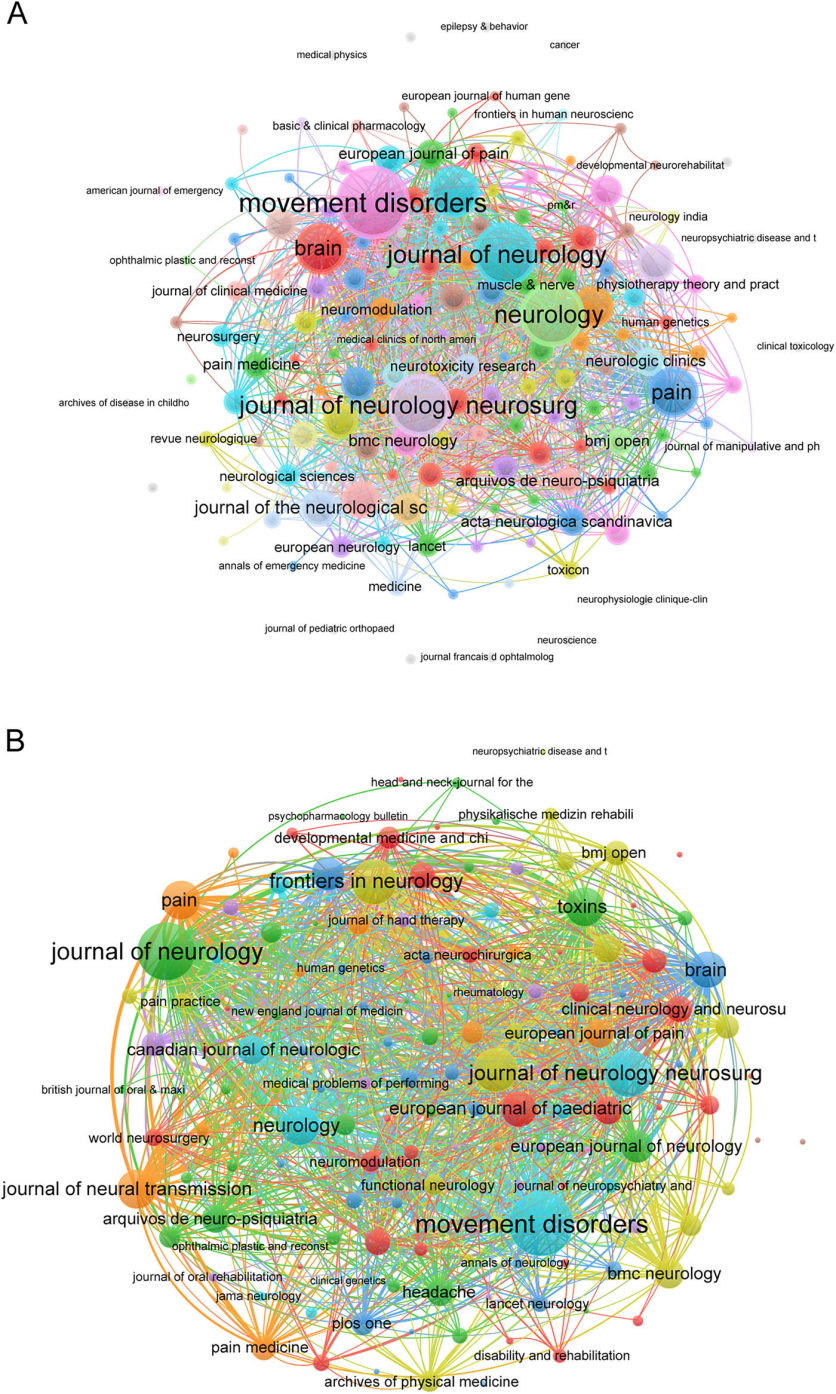
Network analyses of journals. (A) The co‐occurrence networks of journals. The frequency with which journals are cited together within the same articles reflects thematic or topical connections between the researches they publish. Colors indicate different research clusters. (B) The coupling networks of journals. The extent to which journals are linked is based on common references cited in their articles, indicating a shared intellectual foundation or research focus. Colors indicate different research clusters.

Co‐occurrence networks analysis of 161 journals (minimum occurrence threshold = 2) identified three core journals with the highest total link strengths: *Movement Disorders* (link strength = 439), *Neurology* (link strength = 333), and *Journal of Neurology* (link strength = 319) (Figure 6A). In contrast, coupling networks analysis of the same 161 journals (minimum couple threshold = 2) revealed distinct centrality patterns, with *Movement Disorders* demonstrating the strongest connections (link strength = 10,235), followed by the *Journal of Neurology* (link strength = 8415), and the *Journal of Neurology, Neurosurgery And Psychiatry* (link strength = 5557) (Figure 6B).

### 3.6 Analysis of Co‐Occurring Keywords

**FIGURE 7:**
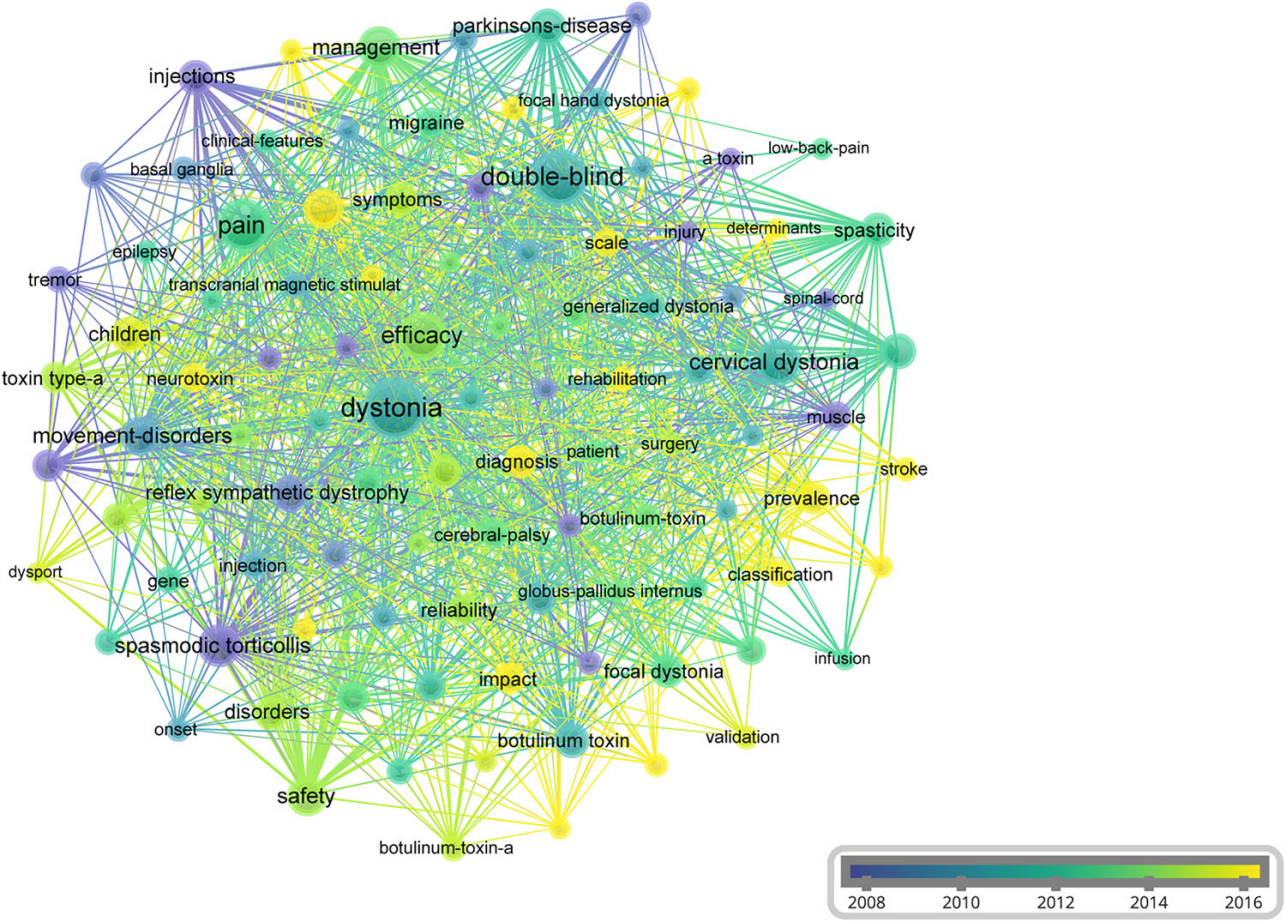
Time‐overlapping co‐occurrence analysis network of keywords. This network visualization displays the co‐occurrence of keywords in selected literature. Each node represents a keyword, with size indicating its frequency of occurrence. Links between nodes represent co‐occurrence in the same documents, with thicker lines showing stronger associations. Link strength measures the frequency of co‐authorship between keywords. Colors reflect the average publication year of the articles, as indicated by the color gradient at the bottom right.

Keyword analysis revealed key research foci and temporal trends within the field. The 20 most frequent keywords, including double‐blind (occurrences = 141), efficacy (occurrences = 103), and spasmodic torticollis (occurrences = 73), were presented in Table 4. Temporal evolution of research themes was visualized through a time‐overlay network map (Figure 7), demonstrating distinct chronological patterns. Early‐stage research (2008–2010, represented by blue nodes) focused on foundational concepts such as “muscle” and “injury.” Mid‐phase investigations (2011–2013, green nodes) showed increased emphasis on clinical trials, particularly “double‐blind” and “efficacy.” Recent advancements (2014–2016, yellow nodes) highlighted emerging priorities in diagnostic refinement and nosological categorization, as evidenced by high‐frequency terms like “diagnosis” and “classification.”

**TABLE 4:** Top 20 keyword co‐occurrence network analysis.

Id	Keyword	Occurrences	Total link strength
628	Double‐blind	141	488
655	Dystonia	168	454
680	Efficacy	103	385
1655	Pain	121	325
352	Cervical dystonia	93	313
2022	Safety	64	268
1902	Quality‐of‐life	64	251
1284	Management	71	233
2148	Spasmodic torticollis	73	233
1412	Movement‐disorders	61	204
2316	Therapy	49	187
1086	Injections	46	178
2156	Spasticity	44	169
1689	Parkinson's‐disease	54	162
368	Children	48	157
599	Disorders	46	151
239	Botulinum toxin	45	148
1970	Reliability	36	142
2353	Toxin type‐a	35	131
564	Depression	38	130

### 3.7 Analysis of Burst Keywords

**FIGURE 8:**
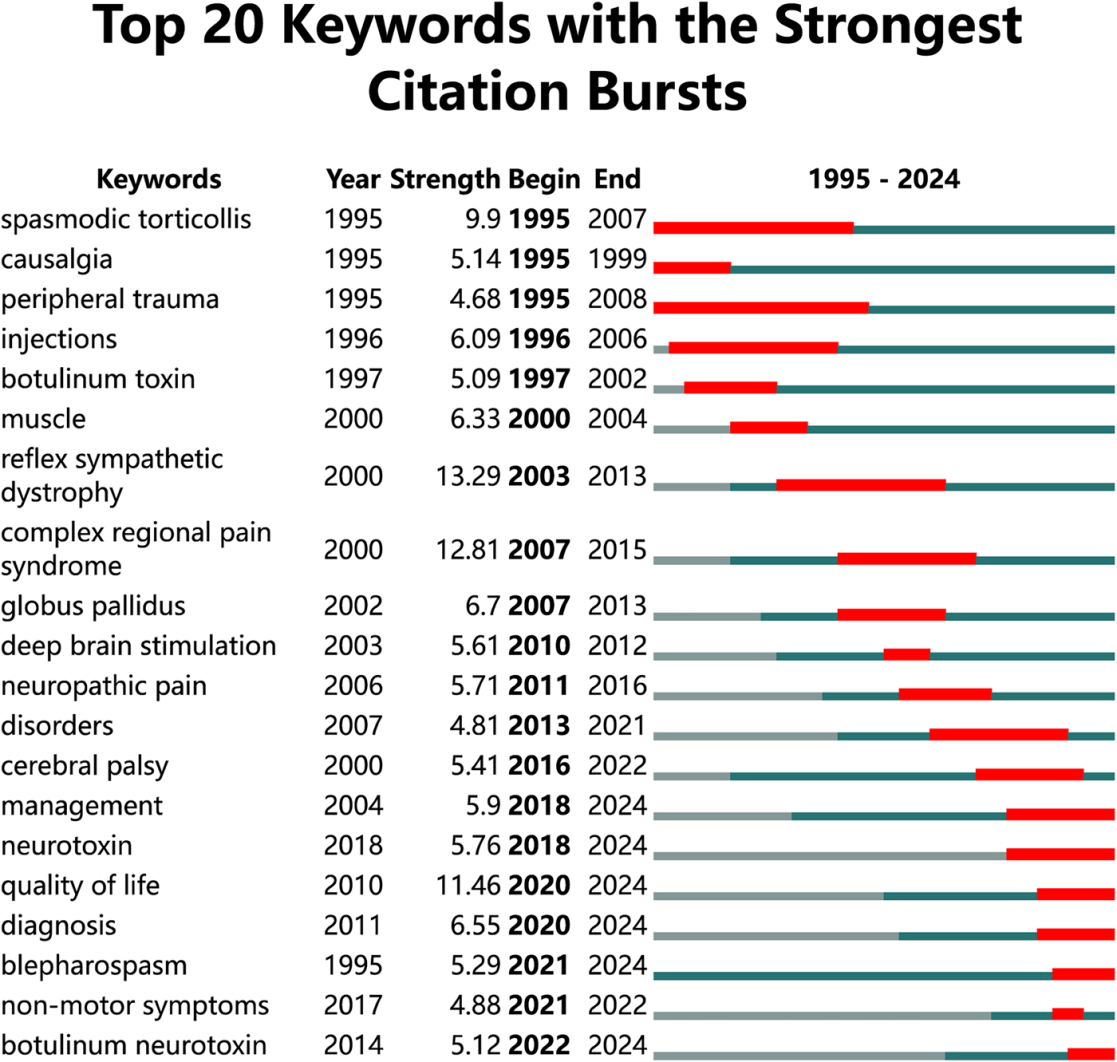
Citation burst analysis of keywords. The blue lines represent the period, and the red lines indicate the burst periods of the keywords.

Citation burst analysis revealed significant temporal shifts in research priorities (Figure 8). Twenty keywords exhibited pronounced citation bursts, with the strongest historical activity observed for “reflex sympathetic dystrophy” (burst strength = 13.29; 2003–2013). Notably, persistent bursts continuing into 2024 highlighted emerging research frontiers, including “blepharospasm,” “botulinum neurotoxin,” “quality of life,” “diagnosis,” “management,” and “neurotoxin.”

## 4 Discussion

### 4.1 Overview of Descriptive Analysis

This bibliometric analysis provides a comprehensive overview of global research trends on pain in dystonia from 1981 to 2025. A total of 1225 articles by 5925 authors were reviewed, involving contributions from 63 countries and 415 journals. The USA dominated in terms of both TP and TC. In terms of institutions, University of London led the publications. Jankovic Joseph emerged as the most impactable contributor with highest TP. High‐impact journals like *Movement Disorders* played central roles in disseminating key findings. Keywords such as “blepharospasm,” “botulinum neurotoxin,” “quality of life,” “diagnosis,” “management,” and “neurotoxin” highlighted the evolving research focus, reflecting a dual focus on refining diagnostic/therapeutic strategies for dystonia and addressing pain management in affected populations.

The USA emerged as the leading contributors to pain in dystonia research, both in terms of publication volume and citations. The dominance of the USA, with institutions such as the Harvard University, reflects its well‐established research infrastructure, significant funding in pain in dystonia research, and leadership in development of neural network biomarker (Valeriani and Simonyan 2020). Rapid growth in Germany can be attributed to its increasing healthcare investment and focus on neurological diseases, including the epidemiology of dystonia (Dressler et al. 2022).

The most impactful contributors, such as Jankovic Joseph, Marinus Johan, and Van Hilten Jacobus J., have significantly shaped the field of pain in dystonia. Jankovic Joseph has made significant contributions to the development of various therapeutic approaches for dystonia, such as dopaminergic therapy, antidopaminergic therapy, anticholinergic therapy, baclofen, botulinum toxin, and others (Jankovic 2013). Meanwhile, Marinus Johan has focused his research on complex regional pain syndrome‐related dystonia, investigating multiple pathological mechanisms (Marinus et al. 2011). Van Hilten Jacobus J., for example, used complex regional pain syndrome as a model to develop concepts on mechanisms of disease that underlie movement disorders like dystonia that occur in response to tissue injury, their assessment, and treatment (van Hilten 2010). Specifically, neurogenic inflammatory responses, nociceptive sensitization, vasomotor dysfunction, and functional and structural changes in central nervous system are the main mechanisms involved in dystonia. These considerations of the multiple mechanisms implicated in the pathophysiology of dystonia provide a basis for biomarker discovery and more targeted therapeutic interventions.

The analysis of journals revealed that *Movement Disorders* and *Neurology* were the most prominent contributors in this field. *Movement Disorders* demonstrated a preference for publishing high‐impact clinical and translational studies on Parkinson and movement disorder diagnosis and treatment (Albanese et al. 2023). The purpose of *Neurology* is to advance the field by presenting new basic research with an emphasis on knowledge that will influence the way neurology is practiced. This distribution indicates that journals with diagnosis and treatment focus are pivotal in shaping the field, while journals with broader scopes contribute to the diversity of research themes.

### 4.2 Research Hotspots

The keyword co‐occurrence network revealed an early fundamental exploration on dystonia (2008–2010), with terms like “injection,” and “injury.” Both traumatic and acquired brain injury can result in diffuse multifocal injury affecting both the pyramidal and extrapyramidal tracts, which may lead to movement disorder, including dystonia (Moon 2022). Pain may be on the same or opposite side of the dystonic muscle posturing, suggesting an origin from either dystonic muscle contraction or from non‐dystonic muscles (e.g., due to passive stretching or active compensatory contraction) (Castagna and Albanese 2019). A large volume of clinical data has demonstrated the efficacy and safety of botulinum toxin type A (BoNTA) injection for management of dystonia and reducing pain (Solish et al. 2021). A recent review and meta‐analysis estimated a mean difference in TWSTRS pain subscore of 2.11 (95% CI: 1.38, 2.83) points for patients treated with BoNTA (any brand) versus placebo at Week 4 (Rodrigues et al. 2020). The best‐known mechanism of BoNTA on pain is the inhibition of acetylcholine release from the presynaptic terminal at the neuromuscular junction with consequent local reduction of muscle fiber activity (Rosales et al. 2021). Relaxation of hyperactive muscles may partly contribute to pain relief through the decompression of the nerve fibers due to the reduction in muscle tone or volume, decreasing afferent activity of spindles and reducing excitability of motoneurons (Marciniec et al. 2019). In addition, a reduction of muscle hypertonicity should provide relief of ischemia, reduction in lactate production, and reduced traction‐related and positional pain (Marciniec et al. 2019).

The middle period (2011–2013) showed increased focus on clinical trials with keywords like “double‐blind,” and “efficacy.” Recently, ASPEN‐1 was a Phase 3, randomized, double‐blind, placebo‐controlled study to evaluate the efficacy, duration of response, and safety of two doses of DaxibotulinumtoxinA for Injection (DAXI), a novel BoNTA formulation in participants with CD (Comella et al. 2024). Improvements were observed for the TWSTRS severity, and pain subscales when compared with placebo at Weeks 4 and 6 (Comella et al. 2024). In addition to drugs, deep brain stimulation (DBS) is another therapeutic strategy. Hock et al. conducted a long‐term open follow‐up of a prospective double‐blind crossover study, where electrodes were bilaterally implanted in both the subthalamic nucleus (STN) and internal pallidum (GPi) in patients with isolated dystonia. Both had a good performance in BFMDRS movement score (Hock et al. 2022). Although originally developed as a treatment for chronic pain, the efficacy of DBS in pain management is still controversial because multicenter trials yielded suboptimal results (Knotkova et al. 2021).

More recent research (2014–2016), themes of “diagnosis,” and “classification” underscored the growing interest of classification of dystonia‐induced pain. Chronic pain is especially prevalent in people with dystonia, which may impact quality of life more significantly than dystonia's motor severity (Listik et al. 2023). It has been suggested that physician often focus on pain management rather than diagnosis and classification of pain, which may further lead to patients experience a delay receiving a diagnosis of dystonia (LaHue et al. 2020). Thus, early diagnosis and classification of dystonia‐induced pain is urgent for this population. Recently, Bruno et al. (2023) developed a new rating instrument, named Pain in Dystonia Scale (PIDS), for pain in adult‐onset idiopathic dystonia and validated it in CD, showing high internal consistency with Cronbach's *α* of 0.9. Unlike the TWSTRS pain subscale, which focuses on the neck, the PIDS provides a comprehensive self‐evaluation of pain from six different body regions, likely encompassing different types of pain. Its total score provides the overall sum of the burden of pain in dystonia, similar to other disease‐specific pain scales (Bruno et al. 2023), addressing the global burden of pain‐related symptoms at the same time in the same patient and its region‐specific burden. Future steps will include validation in other types of dystonia.

### 4.3 Research Frontiers

The keyword burst analysis revealed evolving research frontiers, particularly focusing on emerging keywords in the last five years. The keyword “quality of life” (2020–2024) emphasizes the negative impact of pain caused by dystonia on patients. Similar to other movement disorders, dystonia is a highly stigmatized and debilitating condition (Girach et al. 2019). Furthermore, the quality of life (QoL) of patients with dystonia is not solely determined by motor symptoms; it also includes other symptoms such as abnormal pain perception, cognitive and emotional changes, and sleep disturbances. Research indicates that neuropsychiatric symptoms and pain have a greater impact on patients' quality of life than the severity of dystonia itself (Maione et al. 2023). Furthermore, “Blepharospasm” (2021–2024) as one of the most concerned dystonia forms, is particularly disabling because it interferes with vision, sometimes rendering subjects functionally blind. Its varied presentations are often not appreciated, and it is probably under‐diagnosed. Several reports describe frequent misdiagnoses with initial diagnostic accuracy of 10%∼19%. “Botulinum neurotoxin” (BoNT) (2022–2024) provides an effective treatment option for dystonia and the pain it induces (Rosales et al. 2021). The best‐known mechanism of BoNT action is the inhibition of acetylcholine release from the presynaptic terminal at the neuromuscular junction with consequent local reduction of muscle fiber activity (Trosch et al. 2020). Relaxation of hyperactive muscles may, at least in part, contribute to pain relief through the decompression of the nerve fibers due to the reduction in muscle tone or volume, decreasing afferent activity of spindles and reducing excitability of motoneurons (Marciniec et al. 2019). In addition, a reduction of muscle hypertonicity should provide relief of ischemia, reduction in lactate production, and reduced traction‐related and positional pain. Previous meta‐analytic study included 25 randomized controlled trials and demonstrated significantly lower pain score in the Treatment group with administration of BoNT. Moreover, subgroup analyses showed that BoNT significantly reduced both muscle‐based and non‐muscle‐based pain (Siongco et al. 2020). However, the BoNT also has limitations due to short duration of effects and debate of dosing for pain (Rosales et al. 2021). Looking to the future, clinicians should remain vigilant in tracking their patients' symptoms, including pain, over the course of the injection cycle a look for ways to optimize the therapy plan to maintain symptom relief. The focus of research in the fields of “diagnosis” (2020–2024) and “management” (2018–2024) regarding pain in dystonia continues to emphasize symptom assessment and prognostic management. Currently, there remains room for further improvement in diagnostic methods for symptoms (Jinnah and Factor 2015). In the future, there is a need to develop more effective diagnostic approaches and management strategies to enhance the treatment experience for patients.

This study has several limitations that should be acknowledged. First, the bibliometric analysis was conducted using the WoSCC database, which, while comprehensive, may have excluded relevant studies indexed in other databases such as PubMed or Scopus. This could introduce a selection bias and limit the generalizability of the findings. Second, the analysis was restricted to English‐language publications, potentially overlooking important contributions in other languages. Third, the exclusion of non‐article sources, such as books and editorials, limits the comprehensiveness of the analysis. Finally, the use of author affiliations and collaboration networks may have been affected by inconsistencies in data standardization, such as variations in author names and institutional names across publications. Future studies should consider integrating multiple databases and analyzing broader document types to provide a more comprehensive understanding of research trends in this field.

## 5 Conclusion

This bibliometric study quantitatively assesses the research trends in pain in dystonia from 1981 to 2025, identifying key contributors, research hotspots, and emerging trends. Early research primarily focused on the fundamental exploration of dystonia. Recent research hotspots, however, have targeted the diagnosis, treatment, and management of pain induced by dystonia. These findings highlight the growing interest in this research field to improve patient prognosis through better diagnostic techniques and therapeutic interventions.

## Author Contributions

**Xiaonan Liu**: data analysis and interpretation, manuscript writing, final approval of manuscript. **Bin Wu**: conception and design, administrative support, manuscript writing, final approval of manuscript.

## Ethics Statement

The authors have nothing to report.

## Consent

The authors have nothing to report.

## Conflicts of Interest

The authors declare no conflicts of interest.

## Peer Review

The peer review history for this article is available at https://publons.com/publon/10.1002/brb3.70959

## Supporting information




**Supplementary Materials**: brb370959‐sup‐0001‐Appendix.xlsx

## Data Availability

All data generated or analyzed during this study are included in this article.
